# Case Report: Primary hemophagocytic lymphohistiocytosis with a homozygous PRF1 variant: a case suggesting early immunoporosis and an expanded phenotypic spectrum

**DOI:** 10.3389/fimmu.2026.1875354

**Published:** 2026-06-24

**Authors:** Eman T. Al-Antary, Avanti Gupte, Süreyya Savaşan

**Affiliations:** 1 Division of Hematology/Oncology, Children’s Hospital of Michigan, Detroit, MI, United States; 2 Division of Hematology/Oncology, Pediatric Blood and Marrow Transplantation Program, Barbara Ann Karmanos Cancer Center, Children’s Hospital of Michigan, Detroit, MI, United States; 3 Flow Cytometry Laboratory, Division of Hematology/Oncology, Children’s Hospital of Michigan, Detroit, MI, United States; 4 Department of Pediatrics, Central Michigan University College of Medicine, Mt Clemons, MI, United States; 5 Department of Pediatrics, Wayne State University School of Medicine, Detroit, MI, United States

**Keywords:** HLH - hemophagocytic lymphohistiocytosis, HSCT = hematopoietic stem cell transplant, immunoporosis, pediatric, PRF1 mutation

## Abstract

Familial HLH is an autosomal recessive condition characterized by mutations in genes responsible for the secretory lysosome-dependent exocytosis pathway. Its diagnosis is often challenging to complex clinical presentation, and prompt treatment is essential to avoid fatal outcomes. We describe a novel PRF1 mutation variant p.Y296C c.887 A>G in a pediatric patient with familial HLH (FHL2), which has not yet been reported in the literature to be associated with primary HLH. In this case, we also hypothesize that the phenomenon of immunoporosis contributed to the significant skeletal muscle findings reported in our patient.

## Introduction

Hemophagocytic lymphohistiocytosis (HLH) is a hyperinflammatory syndrome with immune activation comprising a broad disease spectrum, either caused by familial/genetic causes known as primary HLH or as a result of acquired triggers called secondary HLH (1). Its diagnosis is often challenging to complex clinical presentation, and prompt treatment is essential to avoid fatal outcomes. Familial HLH is an autosomal recessive condition characterized by mutations in genes responsible for the secretory lysosome-dependent exocytosis pathway (2). This results in unchecked activation and proliferation of T lymphocytes and macrophages which infiltrate the spleen, liver, bone marrow, lymph nodes, and central nervous system and cause organ damage (3). The earliest reported case of familial HLH was back in 1952 when reportedly two siblings developed fever and hepatosplenomegaly at 9 weeks of age (4). Genetic mutations associated with familial HLH include *PRF1* which encodes perforin, which is the first identified mutated gene causing familial HLH and constitutes one of the most common genetic variants along with UNC13D (5–7).

It has also been noted that there are specific *PRF1* mutations associated with certain ethnic populations, including Middle Eastern, African American, Japanese, and Chinese people (5, 6). We present a novel *PRF1* mutation variant p.Y296C c.887 A>G in a pediatric patient of Middle Eastern descent with familial HLH (FHL2), which has not yet been reported in the literature to be associated with primary HLH.

## Case description

A 17-year-old male of Middle Eastern descent presented with 8 days of fever, night sweats, and headaches. Physical exam was significant for splenomegaly and baseline skeletal changes reflective of thoracic kyphosis and Madelung’s deformity that he was diagnosed 5 years with prior to this presentation. Complete blood count showed pancytopenia with WBC of 1.5×10^9^/L, Hb 7.5g/dL, reticulocytes 5.9%, platelets 49×10^9^/L, absolute lymphocyte count 0.5×10^9^/L, and absolute neutrophil count of 0.7×10^9^/L. Ferritin was 142.6 ng/mL, which may have remained within the normal range because the disease was still in an early phase, prior to the development of substantial cytokine-mediated hyperinflammation; in contrast, triglycerides (191mg/dL), sIL-2R (3,776.6pg/mL), and CXCL9 (6,892 pg/mL) were elevated. Viral testing showed a negative result. Bone marrow was 70% cellular with trilineage hematopoiesis, erythroid hyperplasia, and prominent hemophagocytosis with a normal male karyotype. Due to anemia with reticulocytosis, an inherited anemia panel was obtained, which revealed a heterozygous variant of uncertain significance in the COL4A1 gene (c.3997 G>A [p.Asp1333Asn]); however, *in-silico* analysis implicated a deleterious effect on protein function.

**Figure 1:**
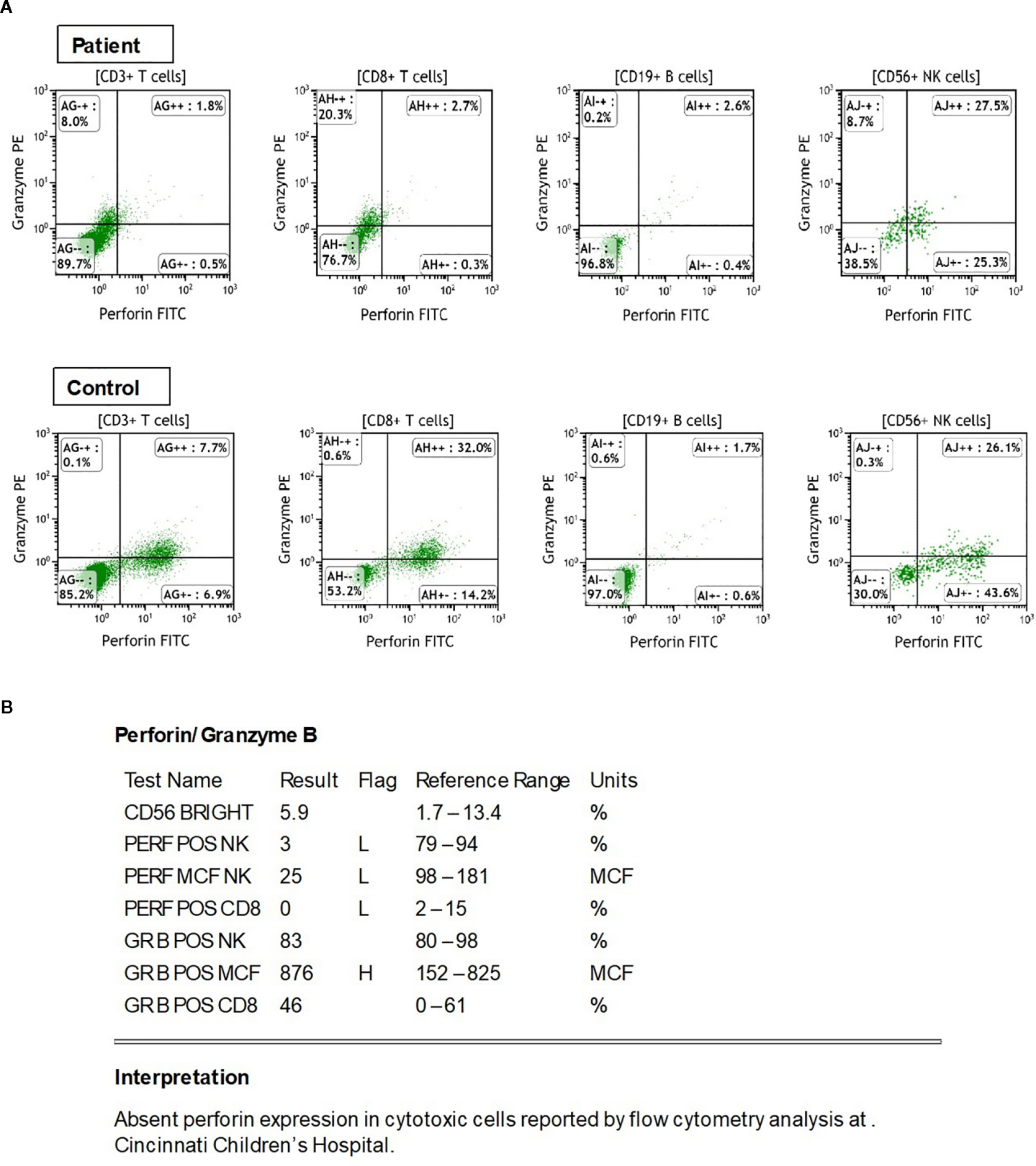
**(A)** In-house flow cytometry analysis showed absent perforin expression on T cells and cytotoxic T cells with a weak expression in some NK cells compared to the control. **(B)** Absent perforin expression in cytotoxic cells reported by flow cytometry analysis at Cincinnati Children’s Hospital.

The patient was seen by the genetics team for investigation of kyphosis and diffuse osteopenia 5 years prior to HLH presentation. A whole exome sequencing did not reveal any causative variants; however, a homozygous mutation in *PRF1* (p.Y296C c.887 A>G) was reported as a variant of uncertain significance at the time. *In-silico* analyses, including protein predictors and evolutionary conservation, supported a deleterious effect. The patient’s mother and father were found to be heterozygous for the Y296C variant in the PRF1 gene. Flow cytometry analysis demonstrated absent perforin expression in natural killer cells, as shown in **Figures 1A, B**. In the context of these genetic findings, together with the patient’s concurrent clinical and laboratory features fulfilling the HLH-2004 diagnostic criteria, the diagnosis of primary HLH attributable to a homozygous *PRF1* mutation was established (1) (**Table 1**).

**Table 1:** HLH-2004 diagnostic criteria.

Criterion	Details
*Genetic diagnosis **OR** 5 out of 8 clinical and laboratory criteria listed below	Molecular diagnosis consistent with HLH
*Fever	Persistent fever (temperature >38.5˚C for > 7 days)
*Splenomegaly	Enlarged spleen (palpable >3cm below costal margin)
*Cytopenias	≥2 cell lines affected: - Hemoglobin <9 g/dL (neonates <10 g/dL) - Platelets <100 ×10^3^/mL - Neutrophils <1 ×10^3^/mL
Hypertriglyceridemia / Hypofibrinogenemia	Triglycerides >265 mg/dL or fibrinogen <150 mg/dL
Hyperferritinemia	Ferritin >500 ng/mL
*Soluble CD25	sCD25 >2400 U/mL or elevated
*Hemophagocytosis	In bone marrow, spleen, lymph nodes, or liver
*NK cell activity	Low or absent NK-cell cytotoxicity

According to the revised diagnostic criteria per HLH-2004 protocol, HLH is diagnosed if either (A) a genetic mutation consistent with HLH is present or (B) our patient meets five out of the eight clinical and laboratory criteria. Asterisks in the figure point out to all the criteria our patient fulfilled (1).

He initially responded well to oral dexamethasone with resolution of his fever and improvement in blood counts and inflammatory markers while completing HLH investigations; however, he experienced an HLH flare during steroid taper. Therefore, he was treated on modified Histiocyte Society HLH 2004 protocol. He experienced significant muscle weakness severely affecting his daily activity and had an episode of aspiration due to significant pharyngeal weakness. As a part of the workup, electromyography was performed and demonstrated moderate motor demyelinating peripheral neuropathy. Hence, his transplant was postponed until he received inpatient rehabilitation. Due to delay in transplant, he was treated on prednisone, etoposide, and cyclosporine due to another short-lived HLH flare to which he responded well.

**Figure 2:**
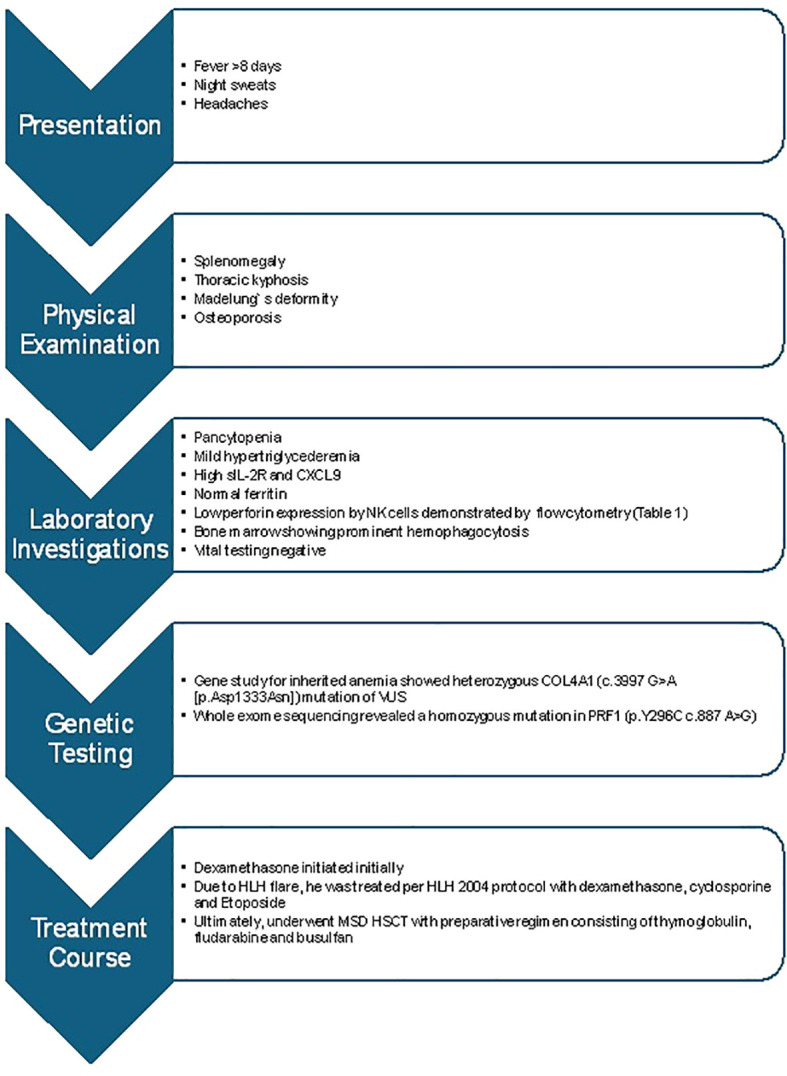
Flow diagram depicting the clinical presentation, laboratory investigations, and treatment course for the patient.

The patient underwent matched sibling donor (MSD) hematopoietic stem cell transplantation (HSCT). His brother was an HLA match with heterozygous *PRF1* mutation. Flow cytometric perforin/granzyme B expression on the brother showed acceptable perforin expression in NK cells. The conditioning regimen was thymoglobulin (2.5 mg/kg/dose on days −8 to −6), busulfan (cumulative AUC over 4 days of 18,000 mmol min), and fludarabine (40 mg/m^2^/dose on days −6 to −3). Graft versus host disease prophylaxis (GvHD) consisted of methotrexate on days +1, +3, +6, and +11 along with tacrolimus from day −3. The initial presentation and clinical course are depicted in **Figure 2**. Currently, 4 years post-HSCT, he has full-donor chimerism with no evidence of HLH flare or GvHD.

## Discussion

To our knowledge, this case represents the first reported homozygous *PRF1* (p.Y296C c.887A>G) mutation associated with primary HLH. *PRF1* mutations underlie FHL type 2 by impairing cytotoxic lymphocyte granule-mediated apoptosis (7). Notably, this mutation was identified more than 5 years prior to clinical disease onset through whole exome sequencing and was initially classified as a VUS. A comprehensive review of publicly available variant databases, including ClinVar and Genome Aggregation Database (gnomAD), was performed to assess the population frequency and prior classification of the PRF1 p.Y296C (c.887A>G) variant. This variant was found to be absent in large population datasets, supporting its potential pathogenicity in accordance with ACMG/AMP criteria (PM2) (8, 9). In addition, several lines of evidence support its clinical relevance. First, the functional role of the PRF1 gene in cytotoxic lymphocyte–mediated apoptosis and its well-established association with primary HLH provide strong biological plausibility (10). Second, the patient’s clinical presentation (meeting 2004 HLH criteria) is highly specific and consistent with PRF1-associated disease (ACMG criterion PP4). Third, the identification of the variant in a homozygous state in the setting of parental consanguinity supports a recessive mode of inheritance and fulfills segregation-based evidence (PM3) (8, 9). Taken together with the demonstrated absence of perforin expression on flow cytometry (functional evidence), these findings support a potential reclassification of this variant from a VUS toward likely pathogenic. However, this interpretation should be considered cautiously and requires confirmation in additional cases, ideally alongside further functional validation studies.

Lymphocyte perforin expression determination by flow cytometry has been shown to facilitate early diagnosis and risk stratification in PRF1-associated HLH (10). In this case, earlier functional testing may have provided diagnostic clarification of the PRF1 variant and prompted closer clinical surveillance, potentially allowing earlier recognition of disease progression and more informed therapeutic decision-making, including consideration of HSCT when clinically indicated—which is an approach associated with improved outcomes in genetically predisposed patients (11).

Another distinctive feature of this case is the presence of marked reticulocytosis and erythroid hyperplasia during active HLH in the absence of thrombotic microangiopathy (TMA). While HLH-associated endothelial injury and secondary TMA have been described, cytokine-driven stress erythropoiesis may also account for these findings (12). For the concomitant identification of the VUS heterozygous *COL4A1* variant, *in-silico* analysis implicated a deleterious effect on protein function, raising the possibility of a modifying role, as *COL4A1* mutations have been linked to vascular fragility, myopathy, and skeletal abnormalities, although hematologic manifestations remain incompletely characterized (13). Whether this mutation influenced erythropoiesis, bone marrow response, or skeletal integrity remains speculative.

Dysfunction of the PRF1 gene, which encodes perforin, results in impaired cytotoxic activity of natural killer (NK) cells and cytotoxic T lymphocytes, a hallmark of primary HLH (7). This defect leads to persistent immune activation characterized by uncontrolled proliferation of activated T cells and macrophages and excessive production of pro-inflammatory cytokines, including interferon-γ (IFN-γ), tumor necrosis factor-α (TNF-α), interleukin-6 (IL-6), and interleukin-17 (IL-17) (14, 15). Beyond their systemic inflammatory effects, these cytokines play a critical role in bone remodeling through the field of osteoimmunology. Specifically, inflammatory cytokines promote osteoclast differentiation and activation via upregulation of the receptor activator of nuclear factor kappa-B ligand (RANKL) pathway, while simultaneously inhibiting osteoblast-mediated bone formation (16–18). Monocytes and macrophages, which are expanded and activated in PRF1 deficiency, serve as osteoclast precursors, further amplifying bone resorption, while activated T cells contribute to increased RANKL expression, reinforcing osteoclastogenesis. Integrative transcriptomic and network-based analyses further support immune–bone crosstalk, identifying immune-related hub genes associated with low bone mass in CD16^+^ monocytes, including cytotoxic lymphocyte–associated genes such as *PRF1* and *SH2D1A* (19).

Within this framework, the concept of “immunoporosis” has been introduced to describe a unified mechanism of bone loss driven by chronic immune activation. Unlike traditional osteoporosis, which is primarily age- or hormone-related, immunoporosis refers to bone fragility arising from sustained inflammatory signaling. In this state, persistent cytokine exposure (particularly TNF-α, IL-6, and IL-17) disrupts the balance between bone formation and resorption by simultaneously enhancing osteoclast activity and inhibiting osteoblast-mediated repair. The result is progressive, inflammation-mediated reduction in bone mass and structural integrity. This concept has been increasingly applied across chronic immune-mediated diseases, highlighting the skeleton as a direct target of immune dysregulation rather than a passive bystander (17, 18, 20).

Importantly, emerging evidence suggests that this state of chronic or subclinical immune activation in PRF1-related disease may precede overt HLH episodes, creating a sustained inflammatory microenvironment that can affect skeletal homeostasis even in early or preclinical stages (14, 21). To our knowledge, skeletal manifestations related to chronic immune activation in PRF1 deficiency have not been well characterized, and this case suggests a potential early osteoimmunologic phenotype preceding overt HLH. Collectively, these findings can support the hypothesis of biologically plausible link between PRF1-associated immune dysregulation and skeletal involvement, suggesting that osteopenia or osteoporosis may represent early or underrecognized manifestations within the phenotypic spectrum of perforin deficiency.

Severe myopathy and demyelinating peripheral neuropathy further complicated the clinical course. Acute glucocorticoid toxicity is a well-recognized cause of bulbar weakness and pharyngeal dysfunction, whereas exposure to tacrolimus and cyclosporine may have contributed to the peripheral neuropathy. HLH itself also represents a plausible etiologic factor, given that macrophage-mediated myelin injury has been reported in this setting (14, 22, 23). Nevertheless, the severity and likely multifactorial nature of the neuromuscular manifestations suggest a more complex pathophysiology. We therefore hypothesize that combined neural, muscular, and vascular injury, potentially related in part to the *COL4A1* variant, contributed additively to the observed phenotype (24).

In summary, this case represents the first reported case of a homozygous PRF1 mutation (p.Y296C, c.887A>G) associated with primary HLH and underscores the importance of early functional characterization of PRF1 variants, close clinical follow-up, and consideration of HSCT when pathogenicity and clinical risk become established. Conceptually, we propose that PRF1 deficiency-related cytotoxic lymphocyte dysfunction might have led to a level of chronic immune activation, which through osteoimmunologic pathways promoted early immunoporosis. However, given the absence of pre-HLH immunologic data, a causal relationship cannot be established. Therefore, the proposed link between PRF1 deficiency and early immunoporosis should be considered hypothesis-generating. This case expands the phenotypic spectrum of PRF1-associated disease and underscores the need for further studies to investigate whether skeletal manifestations may represent an early feature of immune dysregulation in selected patients with primary HLH.

## Data Availability

The raw data supporting the conclusions of this article will be made available by the authors, without undue reservation.

## References

[1] Canna SW Marsh RA . Pediatric hemophagocytic lymphohistiocytosis. Blood. (2020) 135:1332–43. doi: 10.1182/blood.2019000936 32107531 PMC8212354

[2] Sieni E Cetica V Hackmann Y Coniglio ML Da Ros M Ciambotti B . Familial hemophagocytic lymphohistiocytosis: when rare diseases shed light on immune system functioning. Front Immunol. (2014) 5:167. doi: 10.3389/fimmu.2014.00167 24795715 PMC3997030

[3] Goransdotter Ericson K Fadeel B Nilsson-Ardnor S Soderhall C Samuelsson A Janka G . Spectrum of perforin gene mutations in familial hemophagocytic lymphohistiocytosis. Am J Hum Genet. (2001) 68:590–7. doi: 10.1086/318796 11179007 PMC1274472

[4] Farquhar JW Claireaux AE . Familial haemophagocytic reticulosis. Arch Dis Child. (1952) 27:519–25. doi: 10.1136/adc.27.136.519 13008468 PMC1988563

[5] Trizzino A zur Stadt U Ueda I Risma K Janka G Ishii E . Genotype-phenotype study of familial haemophagocytic lymphohistiocytosis due to perforin mutations. J Med Genet. (2008) 45:15–21. doi: 10.1136/jmg.2007.052670 17873118

[6] Lu G Xie ZD Shen KL Ye LJ Wu RH Liu CY . Mutations in the perforin gene in children with hemophagocytic lymphohistiocytosis. Chin Med J (Engl). (2009) 122:2851–5. 20092789

[7] Stepp SE Dufourcq-Lagelouse R Le Deist F Bhawan S Certain S Mathew PA . Perforin gene defects in familial hemophagocytic lymphohistiocytosis. Science. (1999) 286:1957–9. doi: 10.1126/science.286.5446.1957 10583959

[8] Landrum MJ Lee JM Benson M Brown GR Chao C Chitipiralla S . ClinVar: improving access to variant interpretations and supporting evidence. Nucleic Acids Res. (2018) 46:D1062–7. doi: 10.1093/nar/gkx1153 29165669 PMC5753237

[9] Karczewski KJ Francioli LC Tiao G Cummings BB Alfoldi J Wang Q . The mutational constraint spectrum quantified from variation in 141,456 humans. Nature. (2020) 581:434–43. doi: 10.1530/ey.17.14.3 32461654 PMC7334197

[10] Bryceson YT Rudd E Zheng C Edner J Ma D Wood SM . Defective cytotoxic lymphocyte degranulation in syntaxin-11 deficient familial hemophagocytic lymphohistiocytosis 4 (FHL4) patients. Blood. (2007) 110:1906–15. doi: 10.1182/blood-2007-02-074468 17525286 PMC1976360

[11] Marsh RA Vaughn G Kim MO Li D Jodele S Joshi S . Reduced-intensity conditioning significantly improves survival of patients with hemophagocytic lymphohistiocytosis undergoing allogeneic hematopoietic cell transplantation. Blood. (2010) 116:5824–31. doi: 10.1182/blood-2010-04-282392 20855862

[12] La Rosee P Horne A Hines M von Bahr Greenwood T Machowicz R Berliner N . Recommendations for the management of hemophagocytic lymphohistiocytosis in adults. Blood. (2019) 133:2465–77. doi: 10.1182/blood.2018894618 30992265

[13] Meuwissen ME Halley DJ Smit LS Lequin MH Cobben JM de Coo R . The expanding phenotype of COL4A1 and COL4A2 mutations: clinical data on 13 newly identified families and a review of the literature. Genet Med. (2015) 17:843–53. doi: 10.1038/gim.2014.210 25719457

[14] Jordan MB Allen CE Weitzman S Filipovich AH McClain KL . How I treat hemophagocytic lymphohistiocytosis. Blood. (2011) 118:4041–52. doi: 10.1182/blood-2011-03-278127 21828139 PMC3204727

[15] Henter JI Horne A Arico M Egeler RM Filipovich AH Imashuku S . HLH-2004: Diagnostic and therapeutic guidelines for hemophagocytic lymphohistiocytosis. Pediatr Blood Cancer. (2007) 48:124–31. doi: 10.1056/nejmra2314005 16937360

[16] Takayanagi H . Osteoimmunology: shared mechanisms and crosstalk between the immune and bone systems. Nat Rev Immunol. (2007) 7:292–304. doi: 10.1038/nri2062 17380158

[17] Redlich K Smolen JS . Inflammatory bone loss: pathogenesis and therapeutic intervention. Nat Rev Drug Discov. (2012) 11:234–50. doi: 10.1038/nrd3669 22378270

[18] Zhang W Gao R Rong X Zhu S Cui Y Liu H . Immunoporosis: Role of immune system in the pathophysiology of different types of osteoporosis. Front Endocrinol (Lausanne). (2022) 13:965258. doi: 10.3389/fendo.2022.965258 36147571 PMC9487180

[19] Hu B Kong X Li L Dai F Zhang Q Shi R . Integrative analyses of genes associated with osteoporosis in CD16+ Monocyte. Front Endocrinol (Lausanne). (2020) 11:581878. doi: 10.3389/fendo.2020.581878 33551990 PMC7859337

[20] D'Amelio P Sassi F . Osteoimmunology: from mice to humans. Bonekey Rep. (2016) 5:802. doi: 10.1038/bonekey.2016.29 27195109 PMC4870940

[21] Nathan C Ding A . Nonresolving inflammation. Cell. (2010) 140:871–82. doi: 10.1016/j.immuni.2022.03.016 20303877

[22] Boutin B Routon MC Rocchiccioli F Mayer M Leverger G Robain O . Peripheral neuropathy associated with erythrophagocytic lymphohistiocytosis. J Neurol Neurosurg Psychiatry. (1988) 51:291–4. doi: 10.1136/jnnp.51.2.291 3346698 PMC1031548

[23] De Armas R Sindou P Gelot A Routon MC Ponsot G Vallat JM . Demyelinating peripheral neuropathy associated with hemophagocytic lymphohistiocytosis. An immuno-electron microscopic study. Acta Neuropathol. (2004) 108:341–4. doi: 10.1007/s00401-004-0897-0 15243760

[24] Labelle-Dumais C Schuitema V Hayashi G Hoff K Gong W Dao DQ . COL4A1 mutations cause neuromuscular disease with tissue-specific mechanistic heterogeneity. Am J Hum Genet. (2019) 104:847–60. doi: 10.1016/j.ajhg.2019.03.007 31051113 PMC6506795

